# Hyponatremia and Oxidative Stress

**DOI:** 10.3390/antiox10111768

**Published:** 2021-11-04

**Authors:** Benedetta Fibbi, Giada Marroncini, Cecilia Anceschi, Laura Naldi, Alessandro Peri

**Affiliations:** 1 Pituitary Diseases and Sodium Alterations Unit, AOU Careggi, 50139 Florence, Italy; benedetta.fibbi@unifi.it (B.F.); giada.marroncini@unifi.it (G.M.); 2 Endocrinology, Department of Experimental and Clinical Biomedical Sciences “Mario Serio”, University of Florence, AOU Careggi, 50139 Florence, Italy; cecilia.anceschi@unifi.it (C.A.); laura.naldi1@stud.unifi.it (L.N.)

**Keywords:** hyponatremia, osmotic stress, oxidative stress, ROS, senescence, cancer, osteoporosis, neuronal cells homeostasis

## Abstract

Hyponatremia, i.e., the presence of a serum sodium concentration ([Na^+^]) < 136 mEq/L, is the most frequent electrolyte imbalance in the elderly and in hospitalized patients. Symptoms of acute hyponatremia, whose main target is the central nervous system, are explained by the “osmotic theory” and the neuronal swelling secondary to decreased extracellular osmolality, which determines cerebral oedema. Following the description of neurological and systemic manifestations even in mild and chronic hyponatremia, in the last decade reduced extracellular [Na^+^] was associated with detrimental effects on cellular homeostasis independently of hypoosmolality. Most of these alterations appeared to be elicited by oxidative stress. In this review, we focus on the role of oxidative stress on both osmolality-dependent and -independent impairment of cell and tissue functions observed in hyponatremic conditions. Furthermore, basic and clinical research suggested that oxidative stress appears to be a common denominator of the degenerative processes related to aging, cancer progression, and hyponatremia. Of note, low [Na^+^] is able to exacerbate multiple manifestations of senescence and to decrease progression-free and overall survival in oncologic patients.

## 1. Introduction

Hyponatremia, defined as a serum sodium concentration ([Na^+^]) < 136 mEq/L, is the most frequent electrolyte disorder encountered in clinical practice, especially in hospitalized patients and in the elderly [1]. Since hyponatremia is associated with increased morbidity and mortality even in mildly affected patients, it represents an economic burden in terms of hospitalization and health care costs [2,3,4,5,6,7,8]. From this view, a prompt and appropriate correction of this electrolytic imbalance is critical to prevent short- and long-term complications. However, treating hyponatremia is not always perceived as crucial by clinicians, especially when [Na^+^] is only slightly reduced, but it is potentially associated with negative impacts on body functions [9]. For this reason, the comprehension of molecular mechanisms involved in the pathogenesis of symptoms related to hyponatremia might help to raise the awareness about the importance of correcting even chronic and mild reductions of [Na^+^].

## 2. Hyponatremia and Health

Although it has long been thought that persistently but slightly reduced [Na^+^] was completely inconsequential on health, and therefore did not require any correction [1], nowadays chronic hyponatremia is known to have adverse outcomes on several organs and systems [10].

If prolonged over time, the perturbation of internal homeostasis can lead to permanent injuries of biological functions and potentially life-threatening events, as demonstrated by the association of [Na^+^] even mildly below the normal range with increased mortality [4,11,12,13,14]. This correlation was confirmed in cross-sectional studies performed on both inpatient and outpatient cohorts [11,12], and in a meta-analysis including 81 studies and 147,948 participants, which estimated an overall mortality risk ratio of 2.60 (95% confidence interval [CI], 2.31–2.93) in hyponatremic compared to normonatremic patients [3]. A worse prognosis was observed in patients affected by mild hyponatremia and heart, liver, brain, kidney and lung diseases [8,15,16,17], but this electrolyte imbalance was initially considered as a mere marker of disease severity rather than accelerating patient deterioration [18]. In some clinical settings, such as heart failure [19] and cirrhosis [20], inadequate circulation determines a non-osmotic trigger to vasopressin secretion, aimed to preserve blood pressure and circulating volume; moreover, the release of antidiuretic hormone can be stimulated in response to stress and hypothalamic-pituitary-adrenal axis activation [21]. The simultaneous measurement of plasma sodium and copeptin (a molecule co-released with vasopressin) in 6962 patients, revealed a significant association of all cause 30-day mortality with hyponatremia even independently of copeptin (and consequently of vasopressin) levels [22]. 

Besides the presence of a correlation between hyponatremia and mortality, clinical manifestations of chronic hyponatremia also include neurocognitive deficits and bone fractures/osteoporosis. In a cohort of 122 patients admitted to an emergency department and affected by apparently asymptomatic hyponatremia, the frequency of falls was significantly higher than age-matched normonatremic controls [9]; low [Na^+^] was demonstrated to induce gait disturbances similar to ethanol, which were more severe in patients older than 65 years than in younger subjects [23]. These alterations normalized after hyponatremia correction [9]. More recently, the analysis of data from 5435 patients included in the Osteoporotic Fractures in Men Study revealed a significant association between mild hyponatremia and cognitive impairment and decline at the baseline, evaluated by Mini-Mental Status Examination score or Trail Making Test Part B time. The correlation was much stronger for the second test, which measures executive functions (attention and inhibition control, cognitive flexibility, working memory) [24]. Even if dementia may alter vasopressin secretion [25], the maintenance of the association between cognitive impairment and hyponatremia in patients undergoing hemodialysis and peritoneal dialysis supports a mechanism independent of antidiuretic hormone release [26,27]. Bone fractures are more frequent in mild hyponatremic patients than in normonatremic ones [28,29,30], with a higher risk of hospitalization [12,20,31], increased lengths of stay in the hospital and mortality [32]. The increased rate of fractures associated with chronic hyponatremia is not only due to more frequent falls, but also to a higher prevalence of osteoporosis. Epidemiological data highlighted that the increased risk of bone demineralization depends on both severity and duration of the electrolyte imbalance: the lower the serum [Na^+^] is and the more it is maintained over time, the higher the patients risk of developing osteoporosis is [33]. Several studies have now confirmed a strong correlation between a decrease in serum [Na^+^] by as little as 2 to 4 mEq/L below the normal range and osteoporosis and fragility fractures, exceeding the risk related to the use of corticosteroid drugs or smoking [30,34,35,36]. The direct effect of low [Na^+^] on human health and body homeostasis is confirmed by the reversibility of clinical abnormalities secondary to mild and moderate chronic hyponatremia after appropriate correction. A statistically significant association between an increase in serum [Na^+^] in hyponatremic patients and a reduction in mortality, with a calculated odds ratio of 0.57 (95% CI, 0.40–0.81) was demonstrated in a meta-analysis of 15 studies [37]. Treatment of marked hyponatremia with the vasopressin antagonist tolvaptan improved mental health (SALT-1 and SALT-2) [38], psychomotor speed domain (INSIGHT) [39], Timed Up and Go test, nerve conduction velocities and F-wave latencies [40], and gait disturbances [9]. In the INSIGHT trial, the improvement of bone frailty after 22 days of tolvaptan treatment was also demonstrated [39]. Finally, the improvement of bone density was reported in a young man after removal of a vasopressin-secreting esthesioneuroblastoma of the maxillary sinus and normalization of [Na^+^] [41].

Pulmonary diseases are a frequent cause of hyponatremia, occurring in about 30% of patients affected by pneumonia [42]. Retrospective analysis of hyponatremia occurrence in COVID-19 patients during the first pandemic period demonstrated a prevalence of 22.9% at hospital admission, a worse respiratory performance (evaluated as P/F, i.e., the ratio of the partial pressure of oxygen in arterial blood *PaO*_2_ to the inspired oxygen fraction *FiO*_2_), and higher IL-6 levels in hyponatremic rather than in normonatremic hospitalized patients [43]. Since IL-6 is able to induce vasopressin secretion by a direct hypothalamic stimulation and by inducing alveolar basement membrane injury and pulmonary hypoxia and vasoconstriction [44,45,46,47], the pro-inflammatory cytokine may represent the common denominator of both acute respiratory insufficiency and syndrome of inappropriate antidiuresis (SIAD)-related hyponatremia. A very recent metanalysis of 8 studies and 11,493 patients showed a correlation of hyponatremia with COVID-19 poor outcomes (a composite of mortality, prolonged hospitalization and severe COVID-19, defined as severe pneumonia and/or needing intensive care unit support/invasive mechanical ventilation; OR 2.65 [1.89, 3.72], *p* < 0.001; I2: 67.2%, *p* = 0.003), with a 37% sensitivity and 82% specificity; while normal serum [Na^+^] is associated with a 16% post-test probability of a worse prognosis, the presence of hyponatremia increases this probability up to 33% [48]. 

An intriguing and unexpected association was also observed between chronic hyponatremia and overall and progression-free survival in cancer patients. An underlying tumor is responsible for about 14% of hyponatremias [49], whose prevalence in the oncologic setting varies with tumor type and treatment protocols, as well as serum [Na^+^] threshold employed. The most frequent cause of chronic hyponatremia in cancer patients is SIAD, mainly due to ectopic vasopressin secretion by cancer cells [50,51,52,53]. Several clinical evidences reported hyponatremia as an independent, negative prognostic factor in different types of blood and solid tumors (e.g., lymphoma [54], gastrointestinal cancers [55,56], hepatocellular carcinoma [57,58], mesothelioma [59], renal cell carcinoma [60,61], and small cell lung cancer [62,63]), and a concordant improvement of overall and progression-free survival after the appropriate correction of reduced serum [Na^+^] [24], even in patients with extensive and terminal disease [64]. Accordingly, hyponatremia has been proposed as a biomarker able to identify high-risk subjects affected by lung cancer [65].

## 3. Osmotically-Induced Oxidative Stress

The “osmotic theory” was the first one formulated to explain neurologic symptoms associated with low extracellular [Na^+^]. When hyponatremia occurs, the resulting decrease in plasma osmolality (except for the rare cases of non-hypoosmotic hyponatremia) causes water movement into the brain by osmotic gradient, thus causing cerebral oedema [1,66]. The cellular elements most involved in swelling are astrocytes, namely glial cells which are a constituent of the blood-brain barrier and have a fundamental role in maintaining the fluid and electrolyte concentration of the extracellular space in the central nervous system [67]. In the brain, the intracellular/extracellular ionic homeostasis is particularly important, since excitatory and inhibitory synaptic events are driven by ionic gradients, which regulate the resting potential and the discharge pattern of neurons [68]. Sparing neurons from hypoosmolar stress is functional to preserve brain excitability, which is increased both directly (swelling-induced release of excitatory neurotransmitters) and indirectly (restrained diffusion of neurotransmitters and depolarizing agents due to the reduction of extracellular space volume) by swelling [68]. Therefore, an acute decrease in external osmolality determines an initial astrocyte swelling as a result of water movement from the extracellular to intracellular compartment, thus preventing the same phenomenon from occurring in neuronal cells [69] and limiting brain swelling. This first-line defense mechanism is quickly followed by a process known as volume regulatory decrease. This ancient adaptive mechanism, which is able to counteract cell volume alterations and consequently perturbations of cell functions (cell-cycle progression, proliferation, apoptosis, excitability and metabolism) [70], consists in extruding intracellular solutes (electrolytes and organic osmolytes) together with osmotically obligated water [71]. This phenomenon is crucial in the brain, in which the physical restriction of the skull limits the expansion and may determine a life-threatening increase in intracranial pressure. In the first hours, cells mainly lose inorganic ions (first Na^+^ and Cl^−^, then K^+^), as a result of an energy-dependent mechanism based on the activation of the Na^+^-K^+^ ATPase pump (the first signaling pathway of osmotransduction activated by cell swelling), Ca^2+^-dependent and -independent K^+^ channels, K^+^/Cl^−^ co-transporters and volume-sensitive Cl^−^ channels [66,71,72,73]. In cells exposed to sustained hyponatremia, a delayed loss of small organic osmolytes also starts: myoinisotol, betaine, creatine and amino acids (taurine, glycine, aspartate, glutamine and glutamate) are progressively extruded [74], and their efflux is maintained as long as low [Na^+^] persists as an essential adaptive mechanism in chronic hyponatremia. The completion of inorganic solute extrusion within 48 h defines the empirical threshold for acute (<48 h) and chronic (>48 h) hyponatremia [75].

While chronic hyponatremia has been traditionally defined as asymptomatic because of cell volume adaptation, in the last decade several studies demonstrated that even a mild chronic reduction of [Na^+^] may be associated with neurological signs and symptoms, i.e. gait impairment, attention and memory deficit, and increased risk of falls [3,9,28,76,77]. Accordingly, the correction of low [Na^+^] may effectively counteract the reduced cognitive performances observed in hyponatremic patients compared to normonatremic subjects [37,38,76]. The mechanisms that potentially cause these alterations are not completely understood, but the impairment of excitatory neurotransmitters may be involved. As previously mentioned, glutamate is one of the most important organic osmolytes involved in cellular adaptation to hyponatremia [71]. In physiologic conditions, the extracellular glutamate concentration is kept low to avoid an excessive activation of its receptors and glutamate neurotoxicity (GNT), a condition characterized by time-dependent damage of many cell components leading to cell death and prevented through the astrocytic re-uptake mediated by the Na^+^-dependent glial glutamate transporters GLT-1 and GLAST [78]. While the cerebral extracellular concentration of glutamate is increased under acute hypoosmotic conditions [79], its brain content decreases by about 40% after 14 days of sustained hyponatremia in rats [75], thus suggesting an impairment of synaptic excitatory neurotransmission due to chronic hyponatremia. Moreover, it was demonstrated that the sustained reduction of serum [Na^+^] induces gait disturbances and memory impairment in murine models by decreasing astrocytic glutamate re-uptake (through inhibition of GLT-1 and GLAST activities), and consequently long-term potentiation (LTP) at hippocampal synapses [80]. Nowadays, it is well established that GNT is a result of neuronal Ca^2+^ overloading, which is triggered by acute neuronal swelling (the cellular uptake of extracellular Na^+^ and Cl^−^ causes plasma membrane depolarization, and subsequently Ca^2+^ channel opening) and initiates a cascade-like effect leading to cell death [81]. Beyond mitochondria accumulation of Ca^2+^, the generation of Ca^2+^-dependent reactive oxygen species (ROS) (e.g., hydrogen peroxides and superoxides, hydroxyl radicals and oxygen radicals) undoubtedly takes place in GNT [82,83,84,85,86], which is usually associated with marked oxidative stress [87,88]. ROS trigger peroxidative degradation of lipid membranes and modify the redox state of proteins involved in osmotransduction, specifically osmotically-activated tyrosine kinases (ERK1/2, p38, FAK, members of the Src family), which further increase their activity and alter cellular homeostasis [89,90]. Increased ROS formation after exposure to glutamate is divided in two phases: an early ROS production coupled to xanthyne-oxidase activation [91,92], and a later one mostly due to mitochondria as a by-product of glucose metabolism and ATP generation [85,93,94]. Therefore, some authors concluded that in the early GNT, non-mitochondrial ROS generation triggers a cell defense mechanism, while the delayed superoxide production, as well as in apoptosis, occurs secondary to a defect in mitochondrial electron transport and is a result of mitochondrial damage, which acts as a self-propagating process leading to cell dysfunction and death. In particular, the initial oxidative stress could impair mitochondrial energy production and promote depletion of energy stores, thus affecting intracellular homeostatic and protective mechanisms [84].

Beyond neuroactive solutes depletion by neurons, additional mechanisms are hypothesized to explain neurological alterations observed in chronically hyponatremic patients. It is noteworthy that the increased ROS production is also expected to deplete cellular antioxidant defenses, which in turn amplify oxidative stress and radical-mediated injury [95]. As Schultz et al. demonstrated for the first time in vivo, a disturbance of the antioxidant glutathione homeostasis is linked to both excitotoxic neuronal injury and neurodegeneration [96]. Among organic osmolytes, the antioxidants taurine and glutathione are also extruded by neuronal cells in response to extracellular hypoosmolality [97], and the adaptive decrease in their cell content was supposed to make neurons more susceptible to oxidative injury. In fact, glutathione depletion induced by treatment with buthionine sulfoximine or diethylmaleate exacerbates brain injury due respectively to middle cerebral artery ligation in rats [98], and hyperbaric hyperoxia in humans [99]. Using in vivo and in vitro murine and human models, Clark and colleagues demonstrated that brain tissues and cell cultures reduced their content of taurine and glutathione in response to hypoosmolality, and that the depletion was reverted by a slow normalization of serum [Na^+^] [100]. Regarding intracellular functions involved in the reduced availability of antioxidants, the authors observed an osmotically-induced decrease in the synthetic rate of glutathione (whose direct transport across the blood–brain barrier is supposed to be preserved), and an increased release of taurine from cells into the extracellular medium [100]. It has also been suggested that glutathione produced by astrocytes might be a disposal pathway for glutamate, and that decreased synthesis of the antioxidant due to hypoosmolality could exacerbate the injury induced by neurotransmitter accumulation [100,101]. In agreement with a role of the osmotic depletion of antioxidants in the pathogenesis of hyponatremia-related brain injury, the incidence of cerebral infarction in patients with subarachnoid hemorrhage who developed this electrolyte imbalance was significantly higher than in eunatremic subjects [102]. The same mechanism may also play a role in the pathogenesis of the osmotic demyelination syndrome [100].

In addition to inorganic and organic solutes extrusion, activation of phospholipases (particularly the isoforms A2 and D) is an intracellular pathway involved in osmotransduction signaling, as demonstrated by mobilization of arachidonic acid and lysophosphatidylcholine (LPC) in association with hypoosmotic swelling [37,38]. Arachidonic acid contributes to the regulation of K^+^ and Cl^−^ channel activity and organic osmolyte efflux, and similarly to LPC, promotes the generation of ROS [91]. Interestingly, arachidonic acid and ROS were found to inhibit glutamate uptake in astrocytes [103].

**Figure 1:**
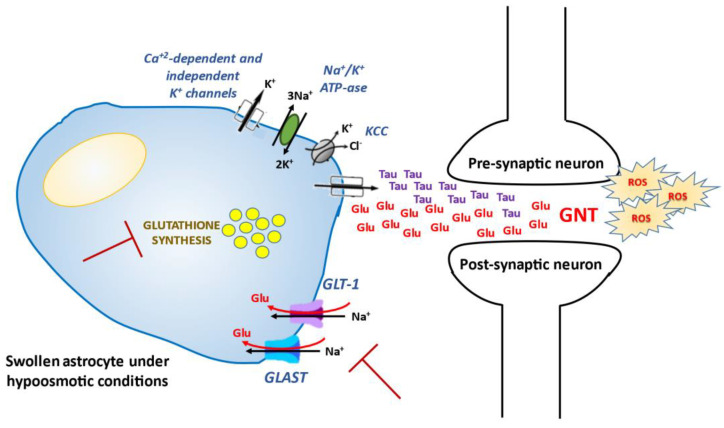
Non osmotically-induced effects of hyponatremia and oxidative stress. GLT-1 and GLAST: Na^+^-dependent glial glutamate transporters; ROS: reactive oxygen species; Glu: glutamate; Tau: taurine; GNT: glutamate neurotoxicity; KCC: K^+^/Cl^−^ co-transporters.

The main mechanisms triggered by hyponatremia and involved in osmotically-induced production of ROS are summarized in Figure 1.

## 4. Non Osmotically-Induced Oxidative Stress

Nowadays, it is well accepted that the central nervous system is not the only target of low [Na^+^]. Indeed, mild chronic hyponatremia has also been associated with detrimental effects on bone, specifically increased risk of osteoporosis and fractures independently of bone demineralization [12,30,31,36,104]. Bone matrix is a large reservoir of the body’s Na^+^, storing approximately one-third of this electrolyte [105]; in dogs, it is an osmotically inactive compartment from which Na^+^ is released during prolonged dietary deprivation [106]. As demonstrated in a rat model of SIAD, hyponatremia-related osteoporosis is due to increased osteoclastic activity, in the absence of other metabolic or hormonal alterations able to explain the accelerated bone resorption (i.e., sex steroid deficiency, metastasis-induced osteolysis, calcium-mediated signals, etc.) [36].

The described detrimental systemic effects secondary to chronic hyponatremia, traditionally defined as an asymptomatic or mildly symptomatic disorder, open a new scenario in understanding the pathophysiology of this condition and its clinical sequelae. In fact, neurological and extra-neurological alterations observed in chronic hyponatremia are explained in principle neither by the “osmotic theory” nor by the homeostatic mechanisms that counteract cell swelling in the presence of extracellular hypotonicity. Therefore, the intriguing hypothesis that hyponatremia could directly impair cellular homeostasis—and hence health status—was postulated. With regard to this point, Barsony et al. first showed that sustained low extracellular [Na^+^] activates osteoclastogenesis and osteoclastic bone matrix resorption in rats both in vivo and in vitro, independently of reduced osmolality [104]. In their view, this response is likely necessary to mobilize bone-stored Na^+^ in the attempt to restore normal extracellular [Na^+^]. Moreover, low [Na^+^] is able to stimulate the differentiation of early-stage osteoclast progenitors compared to normonatremic conditions, by increasing their sensitivity to growth factors (in particular M-CSF) through oxidative stress. These findings suggest the existence of a Na^+^-sensing mechanism or receptor on osteoclasts, as hypothesized also in the central nervous system and in the kidney. Interestingly, the authors found that the activity of the Na^+^-dependent vitamin C transporter is inhibited by low extracellular [Na^+^] in a dose-dependent manner, thus resulting in a reduced uptake of ascorbic acid. As well as playing a central role in setting the equilibrium between osteoclastogenenesis and osteoblastic functions, ascorbic acid is also a key scavenger of oxidative stress [107,108]. As expected, reduced ascorbic acid uptake observed in the above-mentioned model of chronic hyponatremia is associated with increased accumulation of ROS in osteoclastic cells and oxidative DNA damage product 8-OHdg in the sera of hyponatremic rats compared to controls, in agreement with the excessive production of free radicals and osteoclastic bone reabsorption observed in other forms of osteoporosis (e.g., estrogen/androgen deficiency and chronic inflammation) [104]. By developing an in vitro model able to mimic chronic hyponatremia, we further assessed the correlation between hyponatremia and bone health and analyzed the second process involved in bone remodeling alongside resorption, namely neoformation of bone matrix. We showed that reduced extracellular [Na^+^] disrupts gene expression, proliferation, migration, and cytokine production in human mesenchymal stromal cells (hMSC) [109], which are precursors of mesodermal cell types (including adipocytes and osteoblasts of bone matrix) exhibiting different degrees of stemness [110]. In post-menopausal osteoporosis and other conditions characterized by bone loss, the bone marrow shows an imbalance between adipogenesis and osteogenesis, with an accumulation of adipose tissue at the expense of the osteoblastic compartment [111]. In our in vitro model, low [Na^+^] impairs osteoblast activity and differentiation of hMSC, which are shifted toward the adipogenic phenotype at the expense of the osteogenic one [109].

Oxidative stress is also a well-recognized mediator of degenerative processes related to senescence, other than osteoporosis [112], especially in the brain [113]. It is then not inconceivable to speculate that chronic hyponatremia might play a direct role in the pathogenesis of degenerative diseases, in particular aging-related multi-organ pathologies, and that its combination with comorbidities in old people might critically weaken the defense against oxidative stress. As a consequence, sustained low [Na^+^] might accelerate the aging process and represent an independent risk factor for the development and progression of age-related infirmities. In fact, the prevalence of hyponatremia increases progressively with aging, and its major impact (in terms of morbidity and mortality) is exerted in the elderly [114]. The link between chronic hyponatremia and senescence is supported by evidence that chronic hyponatremia (also in this case regardless of hypoosmolality) accelerates and exacerbates multiple manifestations of senescence, including osteoporosis, hypogonadism with testicular fibrosis and arrest of spermatogenesis, reduced adiposity, cardiomyopathy with left ventricular hypertrophy and fibrosis, and sarcopenia, in male rats [115]. Consistently with these data, primary cultures of neonatal rat cardiomyocytes exposed to low extracellular [Na^+^] (but compensated hypoosmolality) and hearts isolated from hyponatremic animals showed increased ROS production and intracellular Ca^2+^ concentrations compared to control cells and tissues [116]. This results in a greater vulnerability of cells against oxidative stress and an exacerbation of myocardial injury due to ischemia/reperfusion, as evidenced by significantly larger infarct size and lower left ventricular developed pressure after exposure to global hypoxia in rats with hyponatremia compared to normonatremic ones [116]. Reoxygenation of cells triggers a burst of ROS, and their increment in low Na^+^ conditions may amplify mitochondrial permeability transition pore opening and induce cell death [117]. Swelling and enlargement of mitochondria and destruction of cristae in cardiomyocytes exposed to low [Na^+^] might be the result of increased ROS content, which in turn could be secondary to intracellular Ca^2+^ overload and activation of Ca^2+^-dependent ROS-generating enzymes [118].

**Figure 2:**
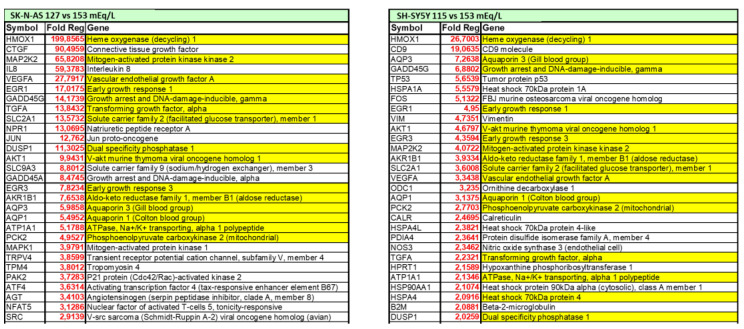
List of differentially expressed genes in two in vitro neuronal models (SH-SY5Y and SKN-AS cell lines), maintained at reduced (115 mmol/L and 127 mmol/L, respectively) or normal (153 mmol/L) [Na^+^], as assessed by microarray analysis. Positive fold-regulations are reported in red, negative fold-regulations are in blue. Yellow marked genes are commonly regulated in both cell lines.

Understanding the potential direct effects of low extracellular [Na^+^] is of particular interest also in the brain, which is one of the main targets of both chronic hyponatremia and senescence. In the last decade, our laboratory demonstrated that low extracellular [Na^+^] directly impairs cellular homeostasis in an in vitro neuronal model of chronic hyponatremia [119]. Sustained low extracellular [Na^+^] was demonstrated to induce cell distress by affecting cell viability and adhesion, expression of anti-apoptotic genes (Bcl-2, DHCR24) and ability to differentiate into a mature neuronal phenotype, even in the presence of compensated osmolality. As a result of a comprehensive microarray analysis, we showed that cell functions involved in “cell death and survival” are the most altered in the presence of reduced [Na^+^] compared to controls, and that the expression of the heme oxygenase-1 (HMOX-1) gene is the most increased [119] (Figure 2).

HMOX-1 is an inducible stress protein with a metabolic function in heme turnover [120] and potent anti-apoptotic and antioxidant activities in different cells, including neurons [121]. In the brain, induction of HMOX-1 by intracellular factors that directly or indirectly generate ROS, preserves neurons from oxidative injury secondary to cerebral ischemia [122] or ethanol intoxication [123]. Elicitation of oxidative stress in the presence of low [Na^+^] was confirmed by cytofluorimetric analysis of total intracellular ROS and ROS-induced lipid peroxidation [124]. These findings reinforce the hypothesis that chronic hyponatremia, through increased oxidative stress and ROS generation, may have a role in brain distress and aging by reducing neuronal differentiation ability, a well-known co-factor in the etiopathogenesis of neurodegenerative diseases such as Alzheimer’s disease [125]. Finally, we also demonstrated that the correction of sustained low extracellular [Na^+^] may not be able to revert all the cell alterations associated with reduced [Na^+^], specifically the expression level of the anti-apoptotic genes Bcl-2 and DHCR24 or of the HMOX-1 gene, even when [Na^+^] was gradually increased [124]. Admittedly, these data appear to reinforce the recommendation to carefully diagnose and treat patients with hyponatremia because a prompt intervention aimed to correct serum [Na^+^] might prevent possible residual abnormalities.

It is now widely accepted that hyponatremia represents a negative independent prognostic factor in oncologic patients, and is associated with poor progression-free and overall survival in several cancers [54,55,56,57,58,59,60,61,62,63]. The direct contribution of this electrolyte imbalance (which cannot be considered a mere surrogate marker of the severity of clinical conditions) is supported by the observation that the correction of serum [Na^+^] may reduce the overall mortality rate in hyponatremic patients [37]. We recently demonstrated, for the first time, that the reduction of extracellular [Na^+^] is able to alter the homeostasis of different human cancer cell lines, thus affecting cell functions (i.e., proliferation, adhesion and invasion) distinctive of a more malignant behavior able to increase cell tumorigenicity [126]. The three steps of carcinogenesis (initiation, promotion, and progression) and the resistance to treatment are strongly impaired by an imbalance between ROS and antioxidant production [127,128]. In fact, oxidative stress regulates cell growth, cytoskeleton remodeling and migration, excitability, exocytosis and endocytosis, autophagy, hormone signaling, necrosis, and apoptosis, namely cell properties deregulated in cancer [127,129]. Furthermore, ROS involvement in carcinogenesis, local invasiveness and metastatization is displayed by their ability to induce genomic instability and/or transcriptional errors [130], and to activate pro-survival and pro-metastatic pathways [129]. Our demonstration of an increased expression of HMOX-1 in cancer cell lines cultured in low extracellular [Na^+^], compared to normal Na^+^ conditions, validates the role of oxidative stress as the molecular basis of hyponatremia-associated poorer outcomes in oncologic patients [126]. Cancer cells have great abilities to adapt to perturbation of cellular homeostasis, including the imbalanced redox status secondary to their high metabolism and local hypoxia. Through a fine regulation of both ROS production and ROS scavenging pathways (the theory of ROS rheostat), they show a high antioxidant capacity, allowing oxidative stress levels compatible with cellular functions even if higher than in normal cells [131]. Recent studies reported an increased expression of ROS scavengers and low ROS levels in liver and breast cancer stem cells [132,133], whose maintenance is crucial for the survival of pre-neoplastic foci. In this view, chemotherapy and radiotherapy, which strongly induce ROS synthesis, are often able to eliminate the bulk of cancer cells but not to definitely cure cancer, because of the up-regulated levels of antioxidants in stem cells, which are thus spared and selected for in the presence of high ROS. An additional mechanism responsible for therapeutic failure is ROS-dependent accumulation of DNA mutations, leading to drug resistance [131]. In this very complex scenario, antioxidant inhibitors are considered a promising therapeutic tool in cancer treatment, especially regarding glutathione metabolism. Since glutathione is a key regulator of the redox balance and protects cancer cells from stress due to hypoxia and nutrient deficiency in solid tumors, the combination of glutathione inhibitors with radiotherapy or chemotherapy could improve the effects of radiation or drugs. However, other enzymes with a scavenging effect on oxidative stress (HSP90, thioredoxin, enzyme poly-ADP-ribose polymerase or *PARP*) may be targeted for anticancer treatments, and are currently under study [131]. Since cancer-related hyponatremia adversely affects the response to chemotherapy and everolimus in metastatic renal cell carcinoma [61,134], correction of low serum [Na^+^] may exert its role in improving cancer survival [135,136] by regulating cancer cell ROS rheostat.

**Figure 3:**
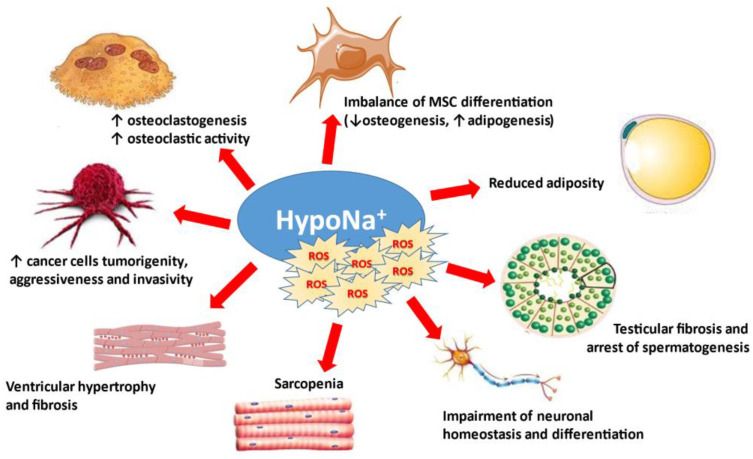
Osmotically-independent effects of hyponatremia and oxidative stress. MSC: mesenchymal stromal cells.

The direct effects of reduced extracellular [Na^+^] on cells and tissues are summarized in Figure 3.

## 5. Conclusions

**Figure 4:**
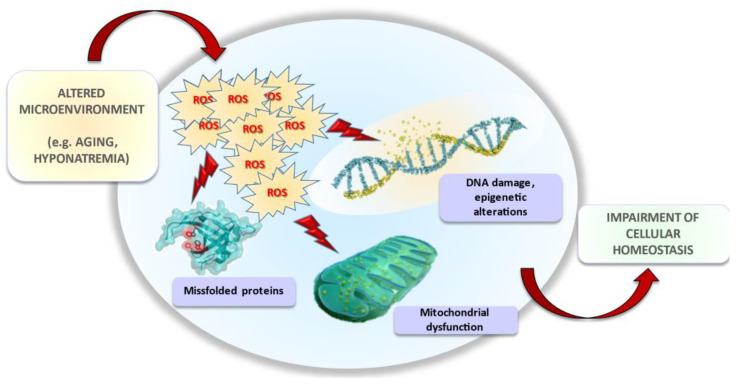
Effects of hyponatremia on cell and tissue homeostasis.

In the last decade, several in vitro and in vivo studies suggested that neurological and extra-neurological symptoms observed in hyponatremic patients might be dependent on a perturbation of cellular homeostasis. Specifically, low extracellular [Na^+^] impairs cellular functions (e.g., gene protein expression, proliferation, migration, and invasion) involved in senescence and carcinogenesis, thus amplifying tissue injuries related to aging and promoting cancer progression. In this scenario, oxidative stress seems to be the common denominator of intracellular events common to both processes (Figure 4). The studies published in recent years opened a new, unpredicted scenario. Further data will be necessary to fully elucidate the specific molecular pathways triggered by reduced extracellular [Na^+^] and responsible for oxidative damage, and to comprehensively understand all potential implications of long-term exposure to hyponatremic conditions.
